# The effects of *Moringa peregrina* seed meal, autoclaving, and/or exogenous enzyme cocktail on performance, carcass traits, meat quality, and blood lipids of broilers

**DOI:** 10.3389/fvets.2023.1158468

**Published:** 2023-07-05

**Authors:** Mohammed A. Al-Harthi, Youssef A. Attia, Mohamed F. Elgandy, Fulvia Bovera

**Affiliations:** ^1^Department of Agriculture, Faculty of Environmental Sciences, King Abdulaziz University, Jeddah, Saudi Arabia; ^2^Department of Veterinary Medicine and Animal Production, University of Napoli Federico II, Napoli, Italy

**Keywords:** *Moringa peregrina* seed meal, autoclaving, enzyme cocktail, broilers, performance, body metabolism

## Abstract

The effects of *Moringa peregrina* seed meal (MPSM), autoclaving, and/or enzyme cocktail addition on performance, profitability, carcass traits, meat quality, and blood lipids of broilers between 1 and 35 d of age were investigated. Seven experimental diets were employed: the control 0% MPSM, 10% raw MPSM, 10% autoclaved MPSM (at a temperature of 120°C and 1 kg/cm^2^ pressure for 30 min), 10% raw MPSM supplemented with enzymes at 0.1 or 0.2 g/kg feed, and 10% autoclaved MPSM supplemented with the same previous enzymes and doses. Each diet was fed to 8 replicates with 5 broilers in each. At the end of the experiment, 3 broilers from each replicate were randomLy chosen to determine carcass traits, meat quality, and blood lipids. Findings at 35 d of age indicated that all 10% raw MPSM treatments with or without enzymes addition impaired growth, feed conversion (FCR), and profitability (*p* < 0.05), but increased feed intake (*p* < 0.05) and did not affect mortality when compared with the control group. The 10% autoclaved MPSM treatments with or without enzymes addition increased feed intake (*p* < 0.05) when compared with the control group, inducing growth equal to the control group (*p* > 0.05), and improving FCR and profitability. Enzymes addition to raw MPSM did not produce positive effects (*p* < 0.05), and no additive effect was observed when autoclaving and enzymes addition were combined (*p* > 0.05) as compared to the autoclaving group. Carcass traits, meat quality, and blood lipids were not significantly affected by MPSM, autoclaving, and enzymes addition. However, intestine, cecum, and gizzard percentages increased (*p* < 0.05) with all 10% raw MPSM treatments, while all 10% autoclaved MPSM treatments could return these values (*p* > 0.05) to the control group, except with gizzard, which exhibited less improvement. Additionally, all autoclaved groups had lower meat pH measured 24 h postmortem (*p <*0.05) compared to the control group. In conclusion, autoclaved MPSM can be included in broilers’ diets at a 10% level without negative effects on performance, carcass traits, meat quality, and blood lipids. This indicates that autoclaving alone is adequate.

## 1. Introduction

The global search for new ingredients such as agricultural by-products for poultry feed has intensified due to the scarcity the conventional feedstuffs resources resulting from the limitations of water and suitable lands for crop production in arid and semi-arid lands, as well as the effects of crises such as the COVID-19 epidemic and the recent war between Russia and Ukraine. Furthermore, the increase in the population and thus the increased needs for poultry production, which is an important source of animal protein, are also contributing factors. In this regard, Moringa tree products could be used as ingredient in animal feeds. Also, it is noteworthy that *Moringa* products contain bio-active substances that can promote growth (1). Humans have used different plants products as food and medicine for centuries. Similarly, *Moringa*-derived natural medicinal products have also been used as additives for farm animals’ feed (2).

*Moringa peregrina* (MP) is a tropical tree of the family *Moringacea* originating in Asia and Africa (3, 4). It has a wide geographic range, growing sporadically from the Dead Sea area to northern Somalia and inside the Arabian Peninsula to the mouth of the Arabian Gulf (5–8). In Jordan, MP grows naturally in Wadi Feynan, Southern Jordan, and the Dead Sea area (9). In most countries, the *Moringa oleifera* (MO) species from the *Moringaceae* family is commercially cultivated. In this family, MP is the second-most economically significant species (10), with the exception that it has high importance in the Kingdom of Saudi Arabia as it is considered a native tree. However, MP has received little attention for its chemical and nutritional properties when compared to MO (2, 11–13). Generally, the use of *Moringa* species in medicine, food, and water purification is well known (14–16). Thus, using MP as an effective and bioactive-rich ingredient to create fortified food products is of the utmost importance (16). Regarding its characteristics, MP measures 20 to 40 cm in diameter and a maximum of 7 to 12 m in height with long pods that hold 15 to 20 seeds. It is locally known as Al-Yassar or Al-Ban (6). The seeds have important nutritional, medicinal, and economical values; for instance, the extracted seed oil is used for cooking, and the intact seeds are used as ingredient in human food, such as cookies (17) and wheat bread (18, 19), and as laxatives and medicine in livestock feed (20). In addition to the histological observations, the evaluation of biochemical parameters has demonstrated the safety of these seeds for human consumption (4). However, studies have rarely evaluated the protein function characteristics, chemical composition, fatty acids composition, medicinal uses, and physicochemical characteristics of MP seeds. In this context, MP seeds have a high level of protein (24.1%), high levels of essential amino acids, some vitamins, and minerals (21), and high amount of oil, ranging from 42% to 54% (4).

MP has recently gained popularity, and some published studies have investigated its pharmacological activities (22), ability to boost immune defense (23), action against multidrug-resistant bacterial and fungi strains (24), and higher resistance to pests and diseases (25). In this regard, a study conducted by Al-Dabbas (26) investigated the antioxidant activity of the leaves and seeds of MP using different extracts and concluded that they are highly effective for this purpose. Similar results were reported about the high contents of total phenolic and total flavonoids in MP leaves (3, 27). It is noteworthy that MP seeds oil contains 33 compounds that have antioxidant activities; thus, it can be used as an alternative to synthetic antioxidants; in this regard, geijerene was identified as the most abundant compound (33.38%), followed by lonalool (23.36%), caryophyllene oxide (19.28%), n-hexadecane (12.59%), and carvacrol (1.89%) (28). On the other hand, Koheil et al. (14) reported high activity of MP seeds ethanolic and aqueous extracts in scavenging free radicals. Furthermore, previous studies have demonstrated that MP seeds and leaves have antidiabetic properties and are used in folk medicine to treat diabetes (4).

However, the main product derived from MP seeds is oil. MP seed meal (MPSM) is the by-product produced after the oil extraction from the seeds, and it has a high nutritional value and is a promising source of various nutrients (27, 29). MPSM contains 93.7% dry matter, 57.1% crude protein, 2.1% crude oil, 6.17% ash, and 34.7% carbohydrates. Moreover, it contains 127 Ca, 742 K, 23.1 Na, and 225 Mg mg/100 g of macro-elements, also contains 19.5 Fe, 1.48 Cu, and 6.14 Zn mg/100 g of micro-elements. Additionally, protein solubility, as an indirect measurement of protein digestibility, was greater in MPSM than soybean meal (30). On the other hand, it was found that MPSM contains more oil than soybean meal but less carbohydrates, protein, and ash (9, 30, 31).

However, it is noteworthy that the industrial use of MP seeds and meal for human consumption is limited due to the bitter taste (21). On the other hand, it was noted that MPSM contains antinutritional agents such as high fibers (non-starch polysaccharides, NSPs); also, phytic acid, tannins, oxalate, alkaloids, phenols, lectins, saponins, cyanogenic glucosides, and chlorogenates; additionally, enzymes inhibitors such as (α-amylase, trypsin, and α-chymotrypsin inhibitors). These antinutritional agents have negative effects on the digestion and utilization of the feed which finally reflect passively on the animal performance (29, 32–36).

It is noteworthy mentioning that the presence of antinutritional agents, also, limits the use of corn and soybean meal-based poultry feeds (37, 38) or shrub-based poultry feeds (39). In this regard, it is very important remembering that antinutritional agents are found in the most, if not all, the ingredients used for poultry feeding. However, the inverse effects of these antinutritional agents depend on their amount and composition in the ingredient/feed and, in turn, their existing in the digestive system of the animal.

Nonetheless, heating the seeds (cooking, roasting, autoclaving) or subjecting them to biochemical procedures (soaking, germination, fermentation) induced significant reduction in antinutrient agents (40). In this regard, the antinutritional agents in black rice were typically reduced or rendered inactive, which also improve protein digestibility when subjected to thermal treatments (41). Furthermore, Al-Harthi et al. (42) found that applying autoclaving technique on untraditional ingredient such as whole *Prosopis juliflora* pods meal (WPPM) which was included in the diet of laying hens at 30% could improve the performance to the level of the control group. They attributed this to the role of autoclaving in weakening the chemical bonds of fiber (NSPs), starch, and protein components in WPPM, and overcoming the negative antinutritional effects by reducing the content, at least, of some substances that cause these negative effects. This, in turn, improved digestion processes and gut ecology, resulting in higher utilization of feed. Also, studies have demonstrated that the processing of watermelon seed flour and sesame seeds by soaking, germination, boiling, and roasting could enhance their nutritional value and quality characteristics, such as color, flavor, texture, antioxidant properties, and shelf life (43, 44). It is noteworthy, to date, most studies that have investigated the effects of soaking, germination and boiling on the antinutritional agents have either been conducted on MO or other types of plants (44).

Nowadays, exogenous enzymes such as amylase, cellulase, xylanase, pectinase, α-galactosidase, β-glucosidase, arabinase, protease, lipase, and phytase or their mixture are being applied extensively to improve the utilization of feed for livestock production. This is due to their similar mode of autoclaving/pelleting actions, thus helping to overcome antinutritional agents and improve digestion processes and gut ecology, leading to better utilization of nutrients which, in turn, improving production and health of broilers and laying hens (42, 45–49). Therefore, the use of enzyme cocktail that contains carbohydrases and proteases is claimed to improve protein, energy, Ca, and P utilization by broilers (50, 51). Using exogenous enzymes that target NSPs, indigestible compounds, might help endogenous digestion by attacking the cell-wall-encapsulated nutrients in poultry diets (49, 52–54). Recently, the addition of enzyme cocktail that acts on NSPs, proteins, and phytates to corn and soybean meal-based diets to improve energy availability for broiler chickens has received significant attention due to its critical role in the degradation of previous nutrients in the intestinal tract. Alqhtani et al. (49) ascertained that appropriately adjusting dietary metabolizable energy and employing enzyme cocktail for NSPs degradation could improve the commercial benefits for producers. On the other hand, when corn-with-cobs meals in 2 forms (mash or pellets) with and without enzyme cocktail addition were included in broilers’ diets, the addition of enzyme cocktail improved feed utilization and growth performance, demonstrating an additive effect of enzyme cocktail addition over pelleting to the extent that their modes of action were comparable (55).

However, there are still conflicting results regarding the beneficial effects of enzyme cocktail addition on poultry performance, which could be attributed to dietary composition, type and age of chicks, type of enzyme/s, the dose given, and the duration of usage, highlighting the need for further research (42, 45–48, 56).

It is noteworthy, to date, most previous studies have investigated MO seed meal as ingredient in poultry feeds. However, to the best of our knowledge, the previous studies have not discussed the use of MPSM as ingredient in poultry feeds. Therefore, this study is of the utmost importance to clarify the possibility of using this by-product in broiler chicken’s feed.

On the other hand, it is worth mentioning that *Moringa oleifera* seed meal was used in the previous studies within the range from 0.1% to 20% in the diets of broilers, laying hens, and Japanese quail, and the most recommendations were between 5% and 10% (57–67). Although it was used successfully at 8.5% and 28% levels in the diets of bocourti catfish (*Pangasius bocourti*) and African catfish (*Clarias gariepinus*) fingerlings when compared to the control groups, respectively (68, 69). Therefore, the 10% level was used in this study. On the other hand, due to the weakness of the digestive system of broilers, especially between 1 and 21 d of age (70, 71), in addition to the expected antinutritional agents in the MPSM as previously mentioned, autoclaving and exogenous enzyme cocktail addition techniques were also applied in this study.

Ultimately, this study aimed to assess the effectiveness of using MPSM, autoclaving, and/or enzyme cocktail addition on the performance, profitability, carcass traits, lymphoid organs, physical and chemical meat quality, and blood lipid metabolites of broiler chickens between 1 and 35 d of age.

## 2. Materials and methods

### 2.1. Ethical approval

This study was conducted at the Hada Al-Sham Research Station, Faculty of Environmental Sciences, King Abdulaziz University, Jeddah, Saudi Arabia. The research included two parts: The first part was the evaluation of MPSM chemical composition. The second part was the feeding experiment that was carried out on 280-1-d-old Ross-308 unsexed broiler chickens. For the feeding experiment, the methodology was verified by the Committee of the Agriculture Department, King Abdulaziz University, regarding the regulations of the scientific research ethics on living creatures. Which controls the rights and welfare of animals according to the Royal Decree No. M/59 dated 24/8/2010, and institutional approve code ACUC-22-1-2.

### 2.2. Feedstuff and chemical analyses

The MP seeds were purchased from Al-Madinah Al-Munawwarah area in Saudi Arabia. The seeds were mechanically pressed, as a cold press, for oil extraction at approximately 40°C, and the by-product MPSM was ground into powder (particle size 0.5 mm); then, it was kept in bags until proximate chemical composition analyses, including moisture (934.01), crude protein (954.01), ether extract (920.39), crude fiber (978.10), ash (942.05), neutral (NDF, 2002:04) and acid (ADF, 989.03) detergent fibers, and acid detergent lignin (ADL, 973.18), calcium (927.02), and phosphorus (964.06) were done following the methods of the Association of Official Analytical Chemists (AOAC) (72).

Furthermore, hemicellulose, nitrogen-free extract (NFE), and organic matter (OM) contents were calculated as follows:

Hemicellulose content = NDF – ADF.

NFE content = [100 - (moisture + crude protein + ether extract + crude fiber + ash)].

OM content = [100 - (moisture + ash)].

The apparent metabolizable energy (AME) value was calculated according to Nascimento et al. (73):

AME = 4,101.33 + 56.28 ether extract − 232.97 ash − 24.86 NDF + 10.42 ADF.

### 2.3. Feeding trial

The feeding assay was carried out using 280 Ross-308 unsexed broiler chickens from 1 to 35 d of age. The broilers were divided into 7 experimental treatments diets (8 replicates per treatment, with 5 broiler chickens per replicate), and housed in metal cages of 1 × 1 m × 60 cm dimensions equipped with 1 tube feeder and 1 nipple waterer each. The chickens were offered 7 experimental mash diets: the control group (the 1st group) was fed a diet free of MPSM, while the other 6 groups fed on diets that included 10% MPSM. In particular, the 2nd group was fed a diet containing 10% raw MPSM, the 3rd group was fed a diet containing 10% autoclaved MPSM (at a temperature of 120°C and 1 kg/cm^2^ pressure for 30 min), the 4th and 5th groups were fed diets containing 10% raw MPSM supplemented with Rovabio Advance PHY T enzyme cocktail at 0.1 and 0.2 g/kg feed, respectively. Finally, the 6th and 7th groups were fed diets containing 10% autoclaved MPSM supplemented with the previous enzyme cocktail and at the same doses. Enzyme cocktail was added to 1 kg feed, and appropriately mixed by small mixer, then this 1 kg feed was added to the total quantity of the feed per treatment which was in a large mixer. The total quantity of feed for each treatment was 160 kg (which means 16 or 32 g enzyme cocktail addition) to ensure that the feed will be more than enough to complete the experiment at 35 d of age.

Rovabio Advance PHY T is an enzymatic cocktail that consists of 20 enzymes, 17 enzymes of them act on NSPs, 2 enzymes act on proteins, and phytase enzyme. However, the producing company provides the activity of the main enzymes, while the remainder enzymes are given the definition “present or existent.” Thus, these are the activities of the main enzymes, endo-1,4-b-xylanase 12,500 VU/g, endo-1,3(4)-b-glucanase 8,600 VU/g, 6-phytase 10,000 FTU/g, endo-1,4-B-glucanase (cellulase) > 1,200 DNS U/g, arabinofuranosidase ≥46,000 ABF U/g. It is produced by Adisseo Company, France. The recommended dose is 100 g/ton of feed.

The chemical analysis of MPSM is presented in Table 1, while the dietary composition and nutrient profiles of the experimental diets are presented in Table 2. The diets were isonitrogenous and isocaloric and formulated according to the National Research Council (NRC) (74). The water and feed were provided *ad libitum* for the whole period of the experiment.

**Table 1:** Chemical composition of *Moringa peregrina* seed meal.

Component	As-fed basis, %
Moisture^a^	2.92
Crude protein^a^	25.3
Ether extract^a^	9.04
Crude fiber^a^	20.1
Neutral detergent fiber^a^	41.5
Acid detergent fiber^a^	29.8
Acid detergent lignin^a^	14.3
Ash^a^	3.56
Ca^a^	0.25
P^a^	0.58
Hemicellulose^b^	11.7
Nitrogen free extract, (NFE)^b^	39.1
Organig matter, (OM)^b^	93.5
Apparent Metabolizable energy, (AME, kcal/kg)^b^	3,060

a Determined.

b Calculated.

Hemicellulose content was calculated as follows = neutral detergent fiber − acid detergent fiber.

NFE content was calculated as follows = [100 − (moisture + crude protein + ether extract + crude fiber + ash)].

OM content was calculated as follows = [100 − (moisture + ash)].

AME was calculated, according to Nascimento et al. (73) as: AME = 4,101.33 + 56.28 EE – 232.97 Ash – 24.86 NDF + 10.42 ADF, (3,060 kcal/kg).

**Table 2:** Composition of the diets^a^.

Ingredient	As-fed basis, %	
*Moringa peregrina* seed meal	0.00	10.0
Corn	53.9	48.0
Soybean meal, 48%	35.9	31.7
Dical phosphate	1.80	1.76
Carbonate calcium	1.20	1.19
Sodium chloride	0.30	0.30
Permix^b^	0.30	0.30
DL-methionine	0.16	0.20
L-cystine	0.04	0.08
L-lysine	------	0.14
Vegetable oil	6.40	6.33
Total	100	100
Determined and calculated analysis, %		
Dry matter^c^	90.8	91.1
ME, kcal/kg^d^	3,200	3,200
CP^c^	22.0	22.0
Methionine^d^	0.50	0.50
Methionine + cystine^d^	0.90	0.90
Lysine^d^	1.20	1.20
Ether extract^c^	8.81	9.38
Linoleic acid^d^	4.98	4.86
Crude fiber^c^	2.59	4.30
Ash^c^	2.95	2.96
Ca^d^	1.00	1.00
Available phosphorus^d^	0.45	0.45

a Diets were composed according to NRC (74).

b The premix provides the following per kilogram of diet: vitamin A 1,700 IU, vitamin D3 7,200 ICU, vitamin E 20 mg, vitamin K 3 mg, vitamin B1 3 mg, vitamin B2 5 mg, niacin 50 mg, pantothenic acid 12 mg, vitamin B6 4 mg, biotin 125 mg, folic acid 1.5 mg, vitamin B12 1.5 mg, choline 1,500 mg, manganese 80 mg, zinc 60 mg, iron 90 mg, iodine 0.40 mg, copper 10.00 mg, selenium 0.20 mg, cobalt 0.15 mg.

c Determined.

d Calculated.

Broiler chickens were raised per standard veterinary prescriptions and husbandry conditions. They were provided with a 23:1 h light–dark cycle, and brooding temperatures were 33, 30, 27, and 24°C during the first, second, third, and then the fourth and fifth weeks of age, respectively, with 60% relative humidity.

### 2.4. Measured criteria

The chicks were weighed based on replicate at 1 (45 ± 2 g), 21, and 35 d of age, and feed intake was also estimated. Feed intake, body weight gain, feed conversion rate (FCR), and mortality rate were calculated using the replicate as the experimental unit. Profitability was calculated using (75):

European Broiler Index (EBI) = (daily weight gain per chicken in gm × survival rate, %) / (feed conversion rate × 10).

At 35 d of age, 2 broilers from each replicate, representing all treatment groups, were randomLy chosen, and slaughtered by Islamic method after being fasted overnight. One broiler from each replicate was used to measure carcass characteristics, where the carcass and the inner and lymphoid organs were separated, weighed, and expressed as a ratio of live body weight. From the slaughtered broilers, 2 breasts from each replicate were using for physical meat quality measurements, while 1 breast and 1 leg from each replicate were using for chemical meat quality measurements. The physical characteristics such as color and pH (measured 24 h postmortem) and shear force were determined following Qaid et al. (76). The color values were gauged by the CIELAB Color System L* (lightness), a* (redness), and b* (yellowness), using Chromameter (model CR-400; Konica Minolta, Tokyo, Japan). The meat pH values were measured using a microprocessor pH meter (model pH 211, Hanna Instruments, Woonsocket, RI, United States). The shear force values were determined using Texture Analyzer (TA-HD; Stable Micro Systems, Godalming, United Kingdom, equipped with a Warner-Bratzler shear blade, which was set at the maximum cutting speed of 200 mm per min, kg/f). Water holding capacity (WHC) was determined using the method of Qaid et al. (76) with a simple modification as follows:

WHC = 100 – [(prepressed meat weight − post-pressed meat weight) / (prepressed meat weight) × 100].

The chemical meat analyses, including moisture, crude protein, ether extract, and ash of the mixed meat (50% breast and 50% thigh) were carried out according to AOAC (72). Glycogen content was calculated as follows:

Glycogen content = 100 – (moisture + protein + ether extract + ash).

All physical and chemical meat quality measurements were done on two samples from each replicate, and the average was calculated.

Additionally at 35 d of age, 8 broilers from each treatment (1 broiler from each replicate) were used to collect blood samples. The samples were collected from the brachial vein (5 mL) and transferred into heparinized tubes. Then, plasma was separated by blood centrifugation at 3,000 × *g* for 15 min at 4°C and stored at −20°C until the analyses of the plasma lipid metabolites were done. The blood lipid components (total triglycerides, total cholesterol, total lipids) were determined by colorimetric methods using commercial diagnostic kits (Diamond Diagnostics Company, United States). According to the following methods, glycerol phosphate oxidase/p-aminophenazone (GPO-PAP) for total triglycerides, cholesterol oxidase/p-aminophenazone (CHOD-PAP) for total cholesterol, and sulfo-phospho-vanillin (SPV) for total lipids.

### 2.5. Statistical analysis

Data were analyzed by the GLM procedure (77), using one-way analysis of variance (ANOVA), considering the replicate as the experimental unit. The means comparison was done using the Least Significant Difference (LSD), and the value of *p <* 0.05 was considered significant. The percentages data were transformed by arcsin (x/100) to improve the normality of data distribution before analysis.

## 3. Results

### 3.1. MPSM chemical composition

As shown in Table 1, the proximate chemical composition of MPSM (as-fed basis, %) contained 2.9 moisture, 25.3 crude protein, 9.04 ether extract, 20.1 crude fiber, 41.5 NDF, 29.8 ADF, 14.3 ADl, 3.56 ash, 0.25 calcium, 0.58 phosphorus, 11.7 hemicellulose, 39.1 NFE, 93.5 OM, and 3,060 kcal/kg AME. The composition of the diets is illustrated in Table 2.

### 3.2. Feed intake, weight gain, FCR, and EBI

The effects of raw MPSM, autoclaved MPSM, and/or enzymes supplementation on performance and EBI of broiler chickens during the first period (1–21 d), the second period (22–35 d), and the overall period (1–35 d) of age are shown in Tables 3–6.

**Table 3:** The effect of *Moringa peregrina* seed meal, autoclaving and/or enzyme supplementation on feed intake (daily and accumulative feed intake per chicken) of broiler chickens during 1–21, 22–35, and 1–35 d of age.

Observations treatments	Daily feed intake 1–21 d, g	Accumulative feed intake 1–21 d, g	Daily feed intake 22–35 d, g	Accumulative feed intake 22–35 d, g	Daily feed intake 1–35 d, g	Accumulative feed intake 1–35 d, g
0% MPSM, the control diet	44.5	935	101^c^	1,414^c^	67.3^c^	2,349^c^
10% raw MPSM	47.7	1,002	124^b^	1,736^b^	78.2^b^	2,738^b^
10% autoclaved MPSM	52.7	1,107	147^a^	2,058^a^	90.4^a^	3,165^a^
10% raw MPSM + EC 0.1 g/kg diet	44.5	935	128^b^	1,792^b^	77.9^b^	2,727^b^
10% raw MPSM + EC 0.2 g/kg diet	46.6	979	132^b^	1,848^b^	80.7^b^	2,827^b^
10% autoclaved MPSM + EC 0.1 g/kg diet	51.1	1,073	150^a^	2,100^a^	90.7^a^	3,173^a^
10% autoclaved MPSM + EC 0.2 g/kg diet	53.0	1,113	148^a^	2,072^a^	91.0^a^	3,185^a^
SEM	4.03	84.7	**4.76**	**66.7**	3.17	110
*p*-value	0.153	0.153	0.0001	0.0001	0.0001	0.0001

MPSM, *Moringa peregrina* seed meal; EC, enzyme cocktail; SEM, standard error of mean; Means within a column not sharing similar superscripts are significantly different (*p* <  0.05).

**Table 4:** The effect of *Moringa peregrina* seed meal, autoclaving and/or enzyme supplementation on growth rate (daily and accumulative weight gain per chicken), and survival rate of broiler chickens during 1–21, 22–35, and 1–35 d of age.

Observations treatments	Daily weight gain 1–21 d, g	Accumulative body weight 1–21 d, g	Daily weight gain 22–35 d, g	Accumulative body weight 22–35 d, g	Daily weight gain 1–35 d, g	Accumulative body weight 1–35 d, g	Survival rate 1–35 d age, %
0% MPSM, the control diet	33.3^a^	699^a^	66.9^a^	936^a^	46.7^a^	1,635^a^	100
10% raw MPSM	21.0^c^	441^c^	46.0^b^	644^b^	31.0^b^	1,086^b^	100
10% autoclaved MPSM	27.7^b^	581^b^	70.9^a^	993^a^	45.0^a^	1,574^a^	100
10% raw MPSM + EC 0.1 g/kg diet	20.6^c^	432^c^	46.2^b^	646^b^	30.8^b^	1,079^b^	100
10% raw MPSM + EC 0.2 g/kg diet	22.8^c^	479^c^	49.8^b^	698^b^	33.6^b^	1,177^b^	100
10% autoclaved MPSM + EC 0.1 g/kg diet	26.8^b^	563^b^	66.9^a^	937^a^	42.9^a^	1,502^a^	100
10% autoclaved MPSM + EC 0.2 g/kg diet	26.9^b^	565^b^	67.2^a^	941^a^	43.0^a^	1,505^a^	100
SEM	1.20	25.1	3.32	44.4	1.62	56.9	-
*p*-value	0.0001	0.0001	0.0001	0.0001	0.0001	0.0001	-

MPSM, *Moringa peregrina* seed meal; EC, enzyme cocktail; SEM, standard error of mean; Means within a column not sharing similar superscripts are significantly different (*p* <  0.05).

**Table 5:** The effect of *Moringa peregrina* seed meal, autoclaving and/or enzyme supplementation on feed conversion rate (daily feed intake per chicken/daily weight gain per chicken) of broiler chickens during 1–21, 22–35, and 1–35 d of age.

Observations treatments	Feed conversion rate 1–21 d	Feed conversion rate 22–35 d	Feed conversion rate 1–35 d
0% MPSM, the control diet	1.34^c^	1.51^c^	1.44^c^
10% raw MPSM	2.27^a^	2.70^a^	2.52^a^
10% autoclaved MPSM	1.90^b^	2.07^b^	2.00^b^
10% raw MPSM + EC 0.1 g/kg diet	2.16^ab^	2.77^a^	2.53^a^
10% raw MPSM + EC 0.2 g/kg diet	2.04^ab^	2.65^a^	2.40^a^
10% autoclaved MPSM + EC 0.1 g/kg diet	1.91^b^	2.24^b^	2.11^b^
10% autoclaved MPSM + EC 0.2 g/kg diet	1.97^ab^	2.20^b^	2.12^b^
SEM	0.123	0.132	**0.100**
*p*-value	0.0001	0.0001	0.0001

MPSM, *Moringa peregrina* seed meal; EC, enzyme cocktail; SEM, standard error of mean; Means within a column not sharing similar superscripts are significantly different (*p* <  0.05).

**Table 6:** The effect of *Moringa peregrina* seed meal, autoclaving and/or enzyme supplementation on European Broiler Index (EBI), (daily weight gain per chicken in gm × survival rate, %)/(feed conversion rate × 10) of broiler chickens during 1–21, 22–35, and 1–35 d of age.

Observations Treatments	EBI 1–21 d	EBI 22–35 d	EBI 1–35 d
0% MPSM, the control diet	249^a^	443^a^	324^a^
10% raw MPSM	92.5^c^	170^c^	123^c^
10% autoclaved MPSM	146^b^	343^b^	225^b^
10% raw MPSM + EC 0.1 g/kg diet	95.4^c^	167^c^	122^c^
10% raw MPSM + EC 0.2 g/kg diet	112^bc^	188^c^	140^c^
10% autoclaved MPSM + EC 0.1 g/kg diet	140^b^	299^b^	203^b^
10% autoclaved MPSM + EC 0.2 g/kg diet	137^b^	306^b^	203^b^
SEM	**14.4**	**25.5**	**13.4**
*p*-value	0.0001	0.0001	0.0001

MPSM, *Moringa peregrina* seed meal; EC, enzyme cocktail; SEM, standard error of mean; Means within a column not sharing similar superscripts are significantly different (*p* <  0.05).

The dietary treatments had no effect on daily or accumulative feed intake (Table 3) during the first 21 d of age (*p* > 0.05), but they had a significant effect (*p* = 0.0001) on daily or accumulative feed intake during the second (22–35 d) or the whole period (1–35 d) of age (*p* < 0.05). It was observed that the inclusion of 10% raw MPSM in the diet increased feed intake during 22–35 d (22.8%) and 1–35 d (16.2%) of age when compared to the control. 10% autoclaved MPSM had an additive effect on the 10% raw MPSM diet, inducing a significant increase (*p* < 0.05) in feed intake during 22–35 d (18.5%) and 1–35 d (15.6%) of age.

During 22–35 d or 1–35 d of age, supplementing the 10% raw MPSM diet with enzymes did not increase feed intake when compared to the 10% raw MPSM diet. The enzymes treatments of the raw MPSM did not have an effect better than that of the 10% raw MPSM diet, or better than or even equivalent to the effect of autoclaved treatment. Additionally, no dose dependence was noted either with the 0.1 or 0.2 g/kg diets during the second and overall periods of age. Furthermore, the enzymes treatments had no additive effect when combined with autoclaved MPSM during the previous periods when compared with autoclaved MPSM treatment alone.

Overall, the dietary treatments had a significant (*p* = 0.0001) effect on the daily and accumulative weight gain of the broilers during 1–21 d, 22–35 d, and 1–35 d of age (Table 4).

When compared to the control, the 10% raw MPSM diet impaired growth during the different periods of age. Autoclaved MPSM treatments with or without enzymes addition improved growth rate significantly (*p* < 0.05) when compared to 10% raw MPSM treatments with or without enzymes supplementation, leading to an increase in daily and accumulative weight gain during the different periods of age. Moreover, in the case of autoclaved MPSM treatments, the broilers demonstrated similar weight gain to the control group during the 22–35 d and 1–35 d of age.

Supplementing the 10% raw MPSM diet with enzymes at both doses (0.1 and 0.2 g/kg diets) did not increase daily or accumulative weight gain when compared to the 10% raw MPSM diet during 1–21 d, 22–35 d, and 1–35 d of age. Like the case of feed intake, the addition of enzymes to the 10% raw MPSM diet did not have a positive effect comparable to autoclaving on daily and accumulative weight gain, and no dose dependence was observed with the 0.1 and 0.2 g/kg diets during the different periods of age. Additionally, no additive effect on daily and accumulative weight gain was observed when autoclaving and enzymes were combined during the different periods of age, indicating that autoclaving alone was adequate. Nonetheless, the dietary treatments had no negative effect on the broilers’ survival rate over the entire experiment.

The dietary treatments had a significant effect (*p* = 0.0001) on the FCR values during the different periods of age (Table 5). FCR was the best with the control group and the worst with the 10% raw MPSM diet group during all periods of age. On the other hand, autoclaving improved FCR (*p* < 0.05) when compared to the 10% raw MPSM diet through all the periods of age.

Supplementing the 10% raw MPSM diet with enzymes did not improve FCR when compared to the 10% raw MPSM diet during the different periods of age. Enzymes treatments were less effective than autoclaving on FCR, and no dose dependence was observed with 0.1 and 0.2 g/kg diets during all periods of age. Moreover, the addition of enzymes had no additive effect on FCR when autoclaving and enzymes were combined compared to the 10% autoclaved MPSM diet during all the periods of age, indicating that autoclaving alone was adequate.

Dietary treatments had a significant effect (*p =* 0.0001) on EBI values during all periods of age (Table 6). Like observations regarding the FCR, the best EBI was recorded with the control group, and the worst was noted in the 10% raw MPSM diet group through all the periods of age. Autoclaving had an additive effect on the 10% raw MPSM diet, improving EBI during all different periods of age.

Supplementing the 10% raw MPSM diet with enzymes did not improve EBI when compared to the 10% raw MPSM diet during the different periods of age. Additionally, no dose dependence with the 0.1 and 0.2 g/kg diets was observed through the different periods of age. Moreover, when autoclaving and enzymes were combined, no additive effect on EBI values were recorded when compared to the 10% autoclaved MPSM diet during all periods of age, indicating that autoclaving alone was adequate.

### 3.3. Carcass traits and immune organs

Tables 7, 8 present the effect of raw MPSM, autoclaved MPSM, and/or enzymes supplementation on the relative weights of dressing, inner organs, and immune organs to the live body weight of broiler chickens at 35 d of age. The previous treatments had no significant effect (*p* > 0.05) on the most ratios of carcass traits and all ratios of lymphoid organs. However, feeding 10% raw MPSM with or without enzymes supplementation resulted in significantly higher ratios (*p* < 0.05) of the intestine, cecum, and gizzard to live body weight when compared to the control group. On the other hand, including 10% autoclaved MPSM with or without enzymes addition in broilers’ diets eliminated these negative effects on the intestine, cecum, (*p* > 0.05) and, to some extent, on the gizzard when compared with the control group. Again, this reinforces that autoclaving alone is adequate.

**Table 7:** The effect of *Moringa peregrina* seed meal, autoclaving and/or enzyme supplementation on dressing and inner organs ratio to live body weight of broiler chickens at 35 d of age.

Observations treatments	Dressing, %	Intestine, %	Cecum, %	Abdominal fat, %	Gizzard, %	liver, %	Heart, %	Pancreas, %
0% MPSM, the control diet	69.4	1.52^b^	0.322^b^	0.395	1.28^c^	2.29	0.576	0.303
10% raw MPSM	63.2	3.01^a^	0.462^a^	0.347	2.47^a^	2.22	0.713	0.298
10% autoclaved MPSM	68.2	2.84^ab^	0.399^ab^	0.329	1.99^b^	2.01	0.602	0.272
10% raw MPSM + EC 0.1 g/kg diet	64.2	3.14^a^	0.461^a^	0.324	2.53^a^	2.37	0.682	0.316
10% raw MPSM + EC 0.2 g/kg diet	68.0	3.13^a^	0.427^a^	0.287	2.29^ab^	2.02	0.618	0.265
10% autoclaved MPSM + EC 0.1 g/kg diet	68.0	2.85^ab^	0.417^ab^	0.296	2.17^ab^	2.28	0.580	0.252
10% autoclaved MPSM + EC 0.2 g/kg diet	67.8	2.97^ab^	0.399^ab^	0.330	2.32^ab^	2.14	0.572	0.263
SEM	7.40	0.231	0.035	0.087	0.136	0.450	0.099	0.045
*p*-value	0.126	0.0009	0.014	0.501	0.0001	0.273	0.345	0.106

MPSM, *Moringa peregrina* seed meal; EC, enzyme cocktail; SEM, standard error of mean; Means within a column not sharing similar superscripts are significantly different (*p* <  0.05).

**Table 8:** The effect of *Moringa peregrina* seed meal, autoclaving and/or enzyme supplementation on immune organs ratio to live body weight of broiler chickens at 35 d of age.

Observations treatments	Spleen, %	Bursa, %
0% MPSM, the control diet	0.078	0.170
10% raw MPSM	0.089	0.164
10% autoclaved MPSM	0.083	0.229
10% raw MPSM + EC 0.1 g/kg diet	0.090	0.190
10% raw MPSM + EC 0.2 g/kg diet	0.083	0.232
10% autoclaved MPSM + EC 0.1 g/kg diet	0.077	0.189
10% autoclaved MPSM + EC 0.2 g/kg diet	0.085	0.132
SEM	0.013	0.038
*p*-value	0.891	0.191

MPSM, *Moringa peregrina* seed meal; EC, enzyme cocktail; SEM, standard error of mean.

### 3.4. Physical quality of breast meat

Table 9 shows the effect of raw MPSM, autoclaved MPSM, and/or enzymes supplementation on the physical quality of breast meat of the broiler chickens at 35 d of age. MPSM, autoclaving, and enzymes treatments had no effect on the color characteristics, WHC, and shear force parameters. However, compared to the control group, feeding broilers 10% autoclaved MPSM with or without enzymes supplementation significantly lowered the pH of breast meat (*p* < 0.05).

**Table 9:** The effect of *Moringa peregrina* seed meal, autoclaving and/or enzyme supplementation on physical breast meat quality of broiler chickens at 35 d of age.

Observations treatments	Color 24 h postmortem			pH 24 h postmortem	Water holding capacity, %	Shear force, kg/f
	L	A	B			
0% MPSM, the control diet	60.0	8.32	4.48	5.55^a^	69.3	1.74
10% raw MPSM	63.0	7.55	3.52	5.45^ab^	63.7	1.66
10% autoclaved MPSM	65.1	5.13	2.24	5.37^b^	62.7	1.67
10% raw MPSM + EC 0.1 g/kg diet	63.2	5.75	3.59	5.45^ab^	63.7	1.54
10% raw MPSM + EC 0.2 g/kg diet	60.6	6.67	2.96	5.47^ab^	65.8	1.58
10% autoclaved MPSM + EC 0.1 g/kg diet	63.7	6.32	2.87	5.40^b^	63.7	1.77
10% autoclaved MPSM + EC 0.2 g/kg diet	65.3	8.19	3.84	5.39^b^	62.5	1.74
SEM	2.99	1.30	0.997	0.330	3.38	0.210
*p*-value	0.087	0.095	0.604	0.0006	0.777	0.236

MPSM, *Moringa peregrina* seed meal; EC, enzyme cocktail; SEM, standard error of mean; Means within a column not sharing similar superscripts are significantly different (*p* <  0.05).

### 3.5. Chemical quality of breast and thigh meat

The effect of raw MPSM, autoclaved MPSM, and/or enzymes supplementation on the chemical quality of mixed breast and thigh meat of broiler chickens at 35 d of age is presented in Table 10. However, all the previous treatments had no significant effect (*p* > 0.05) on water, protein, fat, ash, and glycogen content of the broilers’ meat.

**Table 10:** The effect of *Moringa peregrina* seed meal, autoclaving and/or enzyme supplementation on chemical breast and thigh meat quality of broiler chickens at 35 d of age.

Observations treatments	Meat content as fresh basis, %				
	Water	Protein	Fat	Ash	Mainly glycogen^a^
0% MPSM, the control diet	73.7	21.2	4.03	0.955	0.166
10% raw MPSM	72.6	19.9	6.38	0.970	0.199
10% autoclaved MPSM	73.3	20.4	5.19	0.940	0.223
10% raw MPSM + EC 0.1 g/kg diet	72.8	20.1	6.09	0.953	0.177
10% raw MPSM + EC 0.2 g/kg diet	73.1	20.9	4.81	0.984	0.188
10% autoclaved MPSM + EC 0.1 g/kg diet	72.9	20.8	5.13	0.970	0.201
10% autoclaved MPSM + EC 0.2 g/kg diet	72.9	20.7	5.07	0.966	0.200
SEM	2.31	0.959	1.14	0.037	0.049
*p*-value	0.600	0.673	0.532	0.413	0.434

a Glycogen content was calculated as follows = [100 – (contents of water + protein + fat + ash)]; MPSM, *Moringa peregrina* seed meal; EC, enzyme cocktail; SEM, standard error of mean.

### 3.6. Blood lipid profiles

The effect of raw MPSM, autoclaved MPSM, and/or enzymes supplementation on blood lipid components of the broilers at 35 d of age is displayed in Table 11. Total triglycerides, total cholesterol, and total lipids were not significantly affected (*p* > 0.05) by the different treatments.

**Table 11:** The effect of *Moringa peregrina* seed meal, autoclaving and/or enzyme supplementation on blood lipid components of broiler chickens at 35 d of age.

Observations treatments	Total triglycerides, mg/dL	Total cholesterol, mg/dL	Total lipids, mg/dL
0% MPSM, the control diet	181	197	525
10% raw MPSM	177	194	507
10% autoclaved MPSM	178	201	489
10% raw MPSM + EC 0.1 g/kg diet	180	201	499
10% raw MPSM + EC 0.2 g/kg diet	175	199	511
10% autoclaved MPSM + EC 0.1 g/kg diet	176	200	499
10% autoclaved MPSM + EC 0.2 g/kg diet	178	205	557
SEM	5.25	11.1	32.3
*p*-value	0.449	0.488	0.173

MPSM, *Moringa peregrina* seed meal; EC, enzyme cocktail; SEM, standard error of mean.

## 4. Discussion

### 4.1. MPSM chemical composition

The proximate chemical analysis of MPSM (Table 1) clarifies that it is a beneficial source of nutrients for animals, particularly regarding the dry matter, crude protein (CP), ether extract, ash, NFE, OM, and AME, although it contains high fiber, NDF, ADF, and ADL. Generally, these results align with the previous studies on MPSM (9, 10, 16, 30, 31). Furthermore, its nutrient profiles are similar to those of other *Moringa* species (31, 78, 79). In general, *Moringa* seed meal has nutrient profiles that are better than that of corn but not as rich as that of soybean meal, this is because it contains higher amounts of oil, protein, amino acids, sterols, and polyunsaturated fatty acids when compared with the corn (9, 30, 31, 79–81). However, the chemical analysis of MPSM used in this study demonstrates that it contains better amounts of nutrients than soybean meal (CP 48.5%), except for protein content, where soybean meal contained 88% dry matter, 1.5% ether extract, 27.7% NFE, 81.6% OM, and 2,350 AME (kcal/kg), (82). In line with this trend, similar results were reported about the higher contents of the previous nutrients in MPSM than soybean meal (CP 48.5%), with seeds collected from another area in Saudi Arabi, from Makkah area, and MPSM was obtained by cold press, as done in the present study. This MPSM contained 95.79% dry matter, and as dry matter contained, 27.15% CP, 9.94% ether extract, 30.96% NFE, 96.07% OM, and 3,505 AME (kcal/kg), which was determined by force feeding trial, using 16-wk-old White Leghorn roosters (83). It is worth mentioning that AME content (3,505 kcal/kg) is higher than that calculated by the equation of Nascimento et al. (73) in this study. However, this higher value could be attributed to the age and sex of the animal used in the previous trial (16-wk-old adult Single-Comb White Leghorn roosters), also to the amount and ingredient used (30 g, MPSM only). Zelenka (84) reported that AME values were directly proportional with broilers age (between 12 and 56 d); on the other hand, lower values of AME were noted with increasing feed intake of female chickens (85). It is noteworthy that AME values can be also affected by different factors such as strain/type (meat or layer), diet composition, the inclusion level of specific ingredient, and passage rate through the digestive system (85, 86).

On the other hand, it was noted that the whole MP seeds contain 97.6% dry matter, 24.1% CP, 53.5% ether extract, and 2.6% ash (21), meaning that the MPSM has a chemical composition similar to the whole seed, except for oil content. Ultimately, these values indicate, generally, that *Moringa* seeds and meal are beneficial as food and feed. It is worth mentioning that differences in the chemical composition values of *Moringa* among different studies, if found, can be attributed to the type of *Moringa* species (*peregrina* or *oleifera*), the part of the plant used (seeds, pods, leaves, meal), the environmental conditions and agricultural management of *Moringa* trees, with emphasis, in the case of the meal, on the way of its production (cold or hot extraction).

### 4.2. Feed intake, weight gain, FCR, and EBI

The results regarding performance (feed intake, weight gain, and FCR), and EBI for the first period (1–21 d) indicated that the amount of feed consumed did not differ significantly among all groups, while there were significant differences in the growth rate among these groups. In this regard, the control group demonstrated the best growth rate (*p* < 0.05), and this can be attributed to the quality of the consumed feed, thus enabling broilers to benefit from its nutrients to a great extent. However, groups fed 10% autoclaved MPSM with or without enzymes addition recorded better growth rate (*p* < 0.05) than groups fed 10% raw MPSM with or without enzymes addition. Additionally, groups fed 10% raw MPSM with or without enzymes addition recorded similar growth rate (*p* > 0.05). These findings can be explained by the antinutritional agents found in the raw MPSM (which will be discussed below), and the weakness of the broilers’ digestive system during this stage (70, 71). FCR and EBI values behaved the same trend, which was the best with the control group, followed by the autoclaved MPSM groups, and then the raw MPSM groups.

During the second period (22–35 d) the feed consumption of all groups fed on MPSM increased significantly (*p* < 0.05) than the control group. This might be to compensate their requirements of energy and protein, and the maturity of the digestive system during this stage (70, 71). However, all groups fed autoclaved MPSM consumed more amount of feed than those groups on the raw MPSM, which can be attributed, undoubtedly, to the role of autoclaving. This effect was achieved, mostly, by weakening or dismantling the chemical bonds of MPSM, especially the antinutritional agents as NSPs (crude fiber, NDF, ADF, and ADL), and might be the other components in MPSM such as starch and protein. This consequently induced overcoming or reducing the negative effects of antinutritional agents and, in turn, improved digestion processes and gut ecology that resulted in greater utilization from the MPSM (42). The positive effect of autoclaving technique on laying hens fed whole dried eggs was previously evidenced, where laying rate, egg mass, and FCR were improved comparable to the control group (87).

On the other hand, the higher feed intake by groups fed autoclaved MPSM with or without enzymes addition during this period (22–35 d) induced a similar growth rate as the control group (*p* > 0.05). However, this was not the case with FCR and EBI, which were lower (*p* < 0.05) with autoclaved groups than the control group.

It is noteworthy that the negative effects of feeding raw MPSM with or without enzymes addition persisted during this period (22–35 d); in this regard, these groups did not benefit from the increase in feed intake as the autoclaved MPSM groups did. Where they exhibited lower performance than the control and autoclaved groups in growth rate, FCR, and EBI. Also, the enzymes addition to the raw MPSM groups did not improve the performance when compared with group fed on raw MPSM without enzymes addition.

Overall, the results for the entire period (1–35 d) indicated that the groups fed 10% raw MPSM were negatively impacted in all previous criteria (weight gain, FCR, and EBI) when compared with the control group (*p* < 0.05), except for feed intake, which was higher (*p* < 0.05) than the control group. Moreover, the results demonstrated that using autoclaving technique could reduce these negative effects to the extent that these groups achieved weight gain comparable to the control group (*p* > 0.05). Of course, this was achieved by the compensation which happened through the second period (22–35 d) with these groups fed autoclaved MPSM. However, FCR and EBI values of groups fed on autoclaved MPSM still lower than the control group. Additionally, the results determined that adding enzymes to 10% raw MPSM at both doses (0.1 and 0.2 g/kg diets) did not help in alleviating these negative effects resulting from using 10% raw MPSM when compared to the control or even autoclaved MPSM groups (*p* < 0.05). Furthermore, combining the autoclaving and enzymes did not lead to better results than using autoclaving alone (*p* > 0.05).

Unexpectedly, the addition of the enzymes did not help in improving the utilization of the diets that contained raw MPSM. The enzyme cocktail used in this study contained a variety of active enzymes, and it has been expected to target multiple components in the feed, producing a greater effect than using an individual enzyme that targets a single substrate, inducing an increase in nutrients release; consequently, more nutrients will be available for absorption and utilization (49, 88).

However, several aspects should be clarified about the role of exogenous enzymes and their effects on the utilization of the diets. These aspects are very likely the reasons for the conflicting results regarding the benefit of adding enzymes to feeds in the previous studies (42, 45, 47, 48, 56, 89–91).

These aspects can be summarized in the relationship among three important factors: (1) the chemical composition of the ingredient used in the feed; (2) the specifications of the beneficiary animal; and (3) the specifications and functions of the exogenous enzymes used. The following is a brief elucidation of each factor. The first factor involves the composition and amount of the antinutritional agents found in the ingredient used and its inclusion level in the feed, for example the MPSM used here. In this regard, antinutritional agents, as previously mentioned are: (1) NSPs; (2) phytate, tannins, oxalate, alkaloids, phenols, lectins, saponins, cyanogenic glucosides, chlorogenates; and (3) enzymes inhibitors such as (α-amylase, trypsin, and α-chymotrypsin inhibitors). It might be said that there are 3 classifications of antinutritional agents.

These antinutritional agents, generally, impair the processes of feed digestion and utilization in the digestive system, by preventing the endogenous enzymes from doing their actions through forming viscosity, decreasing bile salt secretion, and inhibiting the digestive system enzymes such as α-amylase, α-glucosidase, trypsin, α-chymotrypsin, and lipase from the binding with reactive sites in villus. They, also binding with nutrients and minerals in the feed forming complex compounds that cannot be absorbed by the villus. Moreover, they can harm the structure and function of the lining cells of the digestive system. Consequently, this leading to reduction in starch, fat, protein, and minerals absorption, animal performance, and finally might induce disease symptoms regarding indigestion (47, 49, 92–97).

The second factor involves the specifications of the animal that will benefit from these exogenous enzymes, including its type, age, and the husbandry circumstances under which the animal is tested (environmental conditions such as temperature, humidity, space, ventilation, etc.). The third factor involves the specifications and functions of the exogenous enzymes used (single or blended enzymes, method of production “from bacteria or fungi,” level of acidity in which it/they potently operate, level of appropriate addition, duration of usage).

It is well known that the commercial enzymes that are used in animals’ feeds usually including (carbohydrases, proteinases, and lipases) which act on starch, protein, fat, and “NSPs, the first classification of antinutrient agents” in the ingredient/feed, which means that they act on the first classification of antinutrients (NSPs). Nonetheless, these exogenous enzymes might include in their enzymatic mixture 2 or 3 other enzymes (phytase, glucosidase, and if found, tannase) that can act on the some substances in the second classification of antinutrients (phytates, glucosides, and tannins, respectively). However, the important question remains: do these exogenous enzymes work on other substances belong to the second classification? Moreover, do these exogenous enzymes act on the third classification?

Overall, these exogenous enzymes do not work on the most substances in the second classification of antinutrients and all antinutrients in the third classification. Furthermore, the enzymes inhibitors in the ingredient used in this study (MPSM) or any other ingredient, might block exogenous enzymes too, as is the case with endogenous enzymes (95). This means that partial or complete paralysis of these exogenous enzymes may occur, and this might be the reason behind the absence of the positive effect from these exogenous enzymes used in this study, in specific, on MPSM and, in general, on the traditional ingredients in the feed as corn and soybean meal, that represent about 80% of the diet composition that contains MPSM.

However, returning to the role of exogenous enzymes, one of the reasons for not activating their benefit in this study, especially regarding their expected role, might be the weakness of the dose used, noting that the dose recommended by the producing company was applied (0.1 g/kg diet), and the double dose (0.2 g/kg diet) was also applied in this study. It seems that these recommended doses might be only effective with conventional ingredients that are used in poultry feeds, as corn and soybean meal, and especially with barley and wheat that have, to some extent, higher contents of fibers.

However, very high doses of exogenous enzymes might be required in the case of using untraditional ingredients that have higher complexity in their chemical composition, which reduces the benefit gained from their nutrients. In line with this trend, Alkassar and Alkassar (98) noted significant improvements in feed intake, weight gain, and FCR with broiler chickens fed on wheat/barley-based diets, during the three stages of age (1–21 d, 22–42 d, and 1–42 d), when they used high doses of Rovabio Excel enzyme cocktail, that produced by the *Penicillium funiculosum Pf* strain, 8/403 (IMI 378536). It is produced by Adisseo Company, France. Where they used the following additions, 1.25, 2.5, 3.75, and 5 g/kg diet, although the recommended dose by the producing company is from 0.05 to 0.50 g/kg diet. On the other hand, it was suggested that higher doses of phytase enzyme can be employed if the ingredient’s phytate content was high to obtain the positive effect (90), especially that the increase in fibers content of any ingredient is associated with an increase in phytic acid (99). However, another study reported a reduction in the relative weight of the pancreas to the live body weight when α-amylase enzyme was added to the feed, indicating internal adaptation of the enzyme secretion to the amount of exogenous enzyme added and the dissociation of the targeted substrate in the feed inside the intestinal lumen (100). Similar results were reported where the secretion of pancreatic amylase and proteases (trypsin and chymotrypsin) decreased when chicks were fed diets that included amylase and protease (101), and this might present yet another challenge.

The groups of broilers that fed on 10% autoclaved MPSM with or without enzymes addition were the ones that benefited from the consumed feed during the second period (22–35 d) among all groups that fed on MPSM. This confirms that the positive influence of the autoclaving technique was the strongest, through its mechanism which previously mentioned. Consequently, these broiler chickens had an increased capability to deal and benefit from the MPSM/feed, as the amount of feed intake, and as the endogenous mechanical and chemical actions, ending with better absorption and utilization of nutrients in the MPSM, which can be noted through FCR values when compared with groups fed raw MPSM. Thus, these groups could achieve growth rate similar to the control group during this period (22–35 d), but not better.

However, adding exogenous enzymes at both doses to 10% autoclaved MPSM did not give better performance than using autoclaving alone. These results ascertained the previous suggestion, mentioned above, that the positive effect on the amount and utilization of feed intake was attributed to autoclaving, through its effect on the first classification of antinutrient agents, rather than its effect on the second and third classifications of antinutrient agents. This assumption is confirmed by the absence of the additive effect of the exogenous enzymes when used with autoclaving. A question here can be asked, how these enzymes can work alone to improve diet utilization and broilers performance, while they failed to do this when combined with autoclaving? This was very clear when enzyme cocktail was added to raw MPSM and did not give any improvement in broilers performance, albeit simple, when compared to those fed raw MPSM without enzymes addition. These findings ascertain that the antinutritional agents, especially, those regarding the second and third classifications “through their role as enzymes inhibitors” that present in the raw MPSM blocked the actions of endogenous and exogenous enzymes (95). However, although not seen in this study, the positive effect of autoclaving on reducing or impairing the second and third classifications of antinutritional agents has been reported in the previous studies (42, 102–104). This might be explained by the differences in the application of autoclaving technique (temperature degree and exposure duration), the nature of the ingredient used regarding the chemical (starch, NSPs, protein, fat, minerals), and might be, the physical composition (form/size of particles), types and levels of antinutritional agents that are existent, the inclusion level used in the feed, and finally the animal specifications and circumstances of the experiment that were applied on the animal (type, age, and experimental conditions). It can be said that the results of any parameter are dependent on the factors that the experiment was subjected to, in other words, the complete design of the experiment.

It is worth remembering that traditional feed ingredients that usually used for poultry (corn and soybean meal) are treated in a similar way to autoclaving before using them in animal feed, through heat processes such as extruding or chemical solvents, so that they can be optimally utilized by the poultry.

Interestingly, autoclaving helped, to some extent, solve the problem; however, it did not eliminate it completely. This is evident by the fact that if the negative effects resulting from all kinds of antinutritional agents were eliminated completely, the growth rate of these 3 groups that fed on 10% autoclaved MPSM with or without enzymes addition would have been better than the control group based on the amount of feed they consumed, which was significantly more than the control. This is what the FCR values demonstrate when comparing these 3 autoclaving groups with the control group. On the other hand, all the previous effects of growth rate and FCR values were reflected on the EBI values. However, no mortality was observed throughout the entire period of the experiment, indicating that the raw MPSM is safe at a 10% level, regarding mortality but not performance.

Ultimately, it seems that the continuous use of thermal techniques should be applied in different ways such as autoclaving, microwaving, and boiling, employing different periods and temperature degrees with or without enzymes addition at different doses. Additionally, involving soaking, fermentation, germination, and milling processes in such techniques is highly important. In other words, scientists must research this subject extensively by testing all the possible ideas that were previously mentioned. This can be done by testing each technique alone using different ways, such as experimenting with autoclaving with different durations and heating, and the same can be done with microwaving, boiling, etc. Then, they can combine different techniques in every possible way, aiming to reach the best solution in terms of time and cost. Of course, all these suggestions and plans should be built on the background knowledge that researchers have established in this field. Moreover, it should be known that each untraditional or by-product ingredient needs special treatment, taking in the account all factors that are involving in the experiment design. This is of the utmost importance for using such untraditional ingredients, which has become a critical issue because of many factors such as population increase, disasters, crises, increasing prices of traditional fodders and transportation and insurance, and the expectation of climatic change, etc. Particularly, each area has some plants that suit its environment and can be relied upon, even if within certain limits.

Furthermore, it might be useful for commercial companies, if possible, to think about producing chemical substances that can deal with antinutritional agents, with emphasis on the second and third classifications, by alleviating their adverse effects, and such substances can be added alongside commercial enzymes. This can reduce time and cost compared to using normal methods such as thermal processes, soaking, germination, fermentation, and milling.

The review by Mukherjee et al. (105) encourages this direction, where it is was noted that when soybean meal was fermented by fungi or bacteria, the fermentation by *Aspergillus* induced the production of enzymes such as hemicellulase, hydrolase, pectinase, protease, amylase, lipase, and tannase, with focusing on the tannase enzyme that is usually not found in exogenous enzymes and can degrade gallotannins and ellagitannins, the 2 types of hydrolyzable tannins (tannins are one of the substances in the second classification of antinutrient agents), ending with producing glucose and gallic or ellagic acid. Additionally, using fungi and bacteria (*Bacillus subtilis, Lactobacillus plantarum, and Bifidobacterium lactis*) for fermentation reduced phytate and trypsin inhibitor (substances found in the second and third classifications of antinutrient agents, respectively), increased small-size peptides (< 15 kD), and promoted the release of amino acids. Moreover, the fermentation by bacteria strains was better than fungi, and this was attributed to the fact that fungi grow slower than bacteria. However, the previous researchers concentrated on some complex factors that affect the efficacy of the fermentation, including solid or submerging fermentation, the microorganism used (fungi or bacteria), and the levels of various nutritional components in the soybean meal. Moreover, Bijina et al. (95) mentioned some possible solutions for producing exogenous enzymes that can be used in feedstuffs and cannot be negatively affected by some of these enzymes’ inhibitors found in the ingredients/feeds. These suggested solutions depend on choosing the suitable type and strain of the microorganism (fungi or bacteria) to produce commercial proteases that can be resistant to specific proteases inhibitors that are found in specific plant/s. Generally, all these findings should seriously encourage commercial companies to seek suitable solutions to deal with these antinutritional agents which might help for using untraditional ingredients more effectively and at higher levels.

### 4.3. Carcass traits and immune organs

Generally, the relative weights of the carcass traits and immune organs demonstrate higher values for the intestine, cecum, and gizzard in groups fed 10% raw MPSM with or without enzymes addition when compared to the control group (*p* < 0.05). This could be attributed to the development of the muscles, mucosa layer, intestine length, and villus height of the digestive system as a result of the digestive system’s need to deal with higher fibers content, and also to their complex chemical composition (NDF, ADF, and ADL in MPSM), necessitating the need for greater mechanical strength to digest and move the digesta from one organ to another through the digestive system (106, 107). These results are further supported by the reduced values observed in the 10% autoclaved MPSM groups with or without enzymes addition. As mentioned before about the role of autoclaving in weakening/dissociating the chemical bonds of the MPSM ingredient, including fibers; thus, this alleviates the mechanical actions required for digestion and movement of digesta through the digestive system, inducing a decrease in the relative weights of these organs. Therefore, no significant differences between the 10% autoclaved MPSM groups and the control group were observed, especially with the intestine and cecum (*p* > 0.05) and, to some extent, with the gizzard. These results are in line with Bournazel et al. (108) who found that the inclusion of rapeseed hulls in broilers’ diets induced higher relative gizzard weight at 21 d, while the inclusion of dehulled seeds lowered this value; and recommended using a suitable level of fibers to get beneficial effects, but it should not exceed this level to avoid negative effects on performance. It is worth noting that fibers can improve the beneficial microbial environment of the digestive system (106, 107); however, it should be highlighted that although the presence of fibers in the feed can improve digestive system health, using them at high levels could lead to negative effects on performance. Nonetheless, fibers can be added to the diet to an appropriate extent that improves digestive system health provided that keeping performance and health of the animal at the normal levels.

On the other hand, it is worthwhile focusing on the review of Samtiya et al. (97), where they reported adverse effects on small intestine of rats when purified lectins (one of antinutrients in the second classification) from soybean or beans were fed, these effects included hypertrophy and damage in the small intestine. This means that the negative effects on the digestive system found with groups fed on raw MPSM might be attributed to the presence of some substances regarding the second classification of antinutrients too. Which gives another possibility for explaining these negative effects, “through synergistic effect with fibers.” If this is the case, it can be expected that the positive effects of autoclaving on fibers led to reduce some of the total negative effects of antinutrients (through its effect on NSPs, as mentioned above), which could indue similar values compared with the control group. Especially that the values of intestine, cecum, and gizzard with autoclaved MPSM groups were between the values of the control group and raw MPSM groups.

On the other hand, the study of EL-Hak et al. (4), clarified that different daily doses of MP dry seeds, which were provided orally to rats for 14 d, did not lead to organ toxicity and histopathological changes and that they are safe for human use. Thus, this supports the previous assumption that the negative effects on the digestive system are dependent on all circumstances of the experiment, as previously mentioned.

Generally, the findings in this study indicate that including 10% autoclaved MPSM in broilers’ diets improved performance and profitability without producing negative effects on carcass traits and immune organs. This also indicates that applying the autoclaving technique alone is adequate.

### 4.4. Physical quality of breast meat

In general, breast acidity values tended to be lower with groups fed on MPSM, proving to be significantly different (*p* < 0.05) with 10% autoclaved MPSM groups with or without enzymes addition compared to the control group. Although these differences are minor, they indicate the presence of good amounts of glycogen which was converted to lactate, especially, through and after slaughter. On the other hand, in line with findings in the current study, Cázares-Gallegos et al. (109) noted lowering in pH values of broilers breast meat, aged from 1 to 40 d, when Mexican oregano oil was increased in their diets (0, 200, 400, 600, 800, and 1,000 mg/kg diet). They attributed this to the role of essential components in the oil, which act as antioxidants, by donating hydrogen ions and, in turn, induced lower pH values. It is worth remembering that MPSM, used in this study, containing 9% oil, which containing 33 antioxidants components, as previously mentioned in the introduction (28). Also, it is worth mentioning that some of these antioxidant’s components in MPSM oil are same to those found in Mexican oregano oil, such as carvacrol, α-terpinene, and ɤ-terpinene. Furthermore, it is interesting to focus on the effect of MPSM on pH values, where it decreased this parameter in all MPSM groups, and was significantly with those groups fed on the autoclaved MPSM when compared to the control group. These findings again confirming the positive effect of autoclaving on the utilization of nutrients in MPSM. However, the pH values found here, also indicate that meat acidity of all groups falls within the normal range of broiler chickens’ meat pH (5.35–6.04) 24 h post-slaughter (110). Moreover, the results showed that the decrease in pH values was coincided with a decrease in WHC values and an increase in pale color (L*) values, although they were not significant. These results are, in general, consistent with previous studies (111–115).

On the other hand, the different treatments did not affect shear force values, indicating that MPSM, autoclaving, and enzymes addition are safe on this parameter too. Hussein et al. (116) reported no significant effect on the shear force values (1.18 and 1.30) of broilers raised from 32 to 48 d of age when enzymes were added (0 vs. 0.5 g/kg) to their diet that contained 3,180 kcal/kg. Additionally, Cázares-Gallegos et al. (109) could not find significant differences on shear force values when Mexican oregano oil was included in the diet of broilers at the levels mentioned above, and the values ranged from 1.13 to 1.73. Moreover, similar to the findings in this study, using orange pulp that contained 10% fiber in broilers’ diets at 50 g/kg between 23 and 42 d of age did not affect color, cooking loss, and shear force values, but pH values 24 h post-mortem decreased when compared with the control group (117), which was attributed to the role of hesperidin antioxidants in the orange pulp through its effect on meat oxidative stability. Furthermore, no significant effects on physical meat quality were reported when broiler chickens between 14 and 21 d of age were fed diets containing distillers dried grains with soluble (DDGS) at 20% level as mash, or mash with steam-heating to 75°C and pelleted, except for a notable effect on lightness (L*) values, which were higher with those on the diet that steam-heated to 75°C and pelleted compared to the control group “the mash group” (118).

### 4.5. Chemical quality of breast and thigh meat

The chemical composition of the mixed breast and thigh meat did not differ between the different dietary groups and the control group, indicating that the inclusion of 10% raw or autoclaved MPSM, with or without enzymes addition had no adverse effects on the meat content of water, protein, fat, ash, and glycogen. Similar results were found with broilers between 1 and 42 d of age when fed diets containing 0%, 6%, 12%, 18%, and 24% DDGS, where no significant differences were observed in the percentages of fat, protein, and ash of the thigh and breast meat (119). On the other hand, Lilly et al. (120) ascertained that feeding broilers, between 28 and 42 d of age different levels of protein (15.5%, 17.1%, 20.2%, and 22.5%, but the same content of energy 3,200 kcal/kg) affected the chemical composition of the thigh meat but not the breast meat, although body weight gain decreased with decreasing protein level in the diets. These researchers noted that the fat content was higher, while the protein and water contents were lower in the group that was fed the lowest protein level compared to the other groups.

However, although no significant differences were observed among the chemical composition values of the different meat fractions in this study, these values indicated a direct correlation between water and protein contents in the control group and 10% raw MPSM group, which, in turn, inversely affected fat contents of these two groups. The direct correlation between water and protein content and their inverse effect on fat content are well proven. Additionally, it is worth mentioning that although weight gains were significantly different at 35 d of age among the different groups in this study, the chemical composition of the meat was not affected. This expectation was built upon the negative effects of antinutritional agents, found especially in the raw MPSM treatments, through their negative effects on the processes of digestion and utilization of nutrients found in the raw MPSM, and in the whole feed that included MPSM. Where these antinutritional agents can interact with the nutrients in the feed (starch, fat, protein, and minerals) making them unavailable to the animal. It seems that animal’s body chemical composition can adapts with the amount and composition of feed intake properly, at least, to some extent (86, 119–122). Knowing that, such adaptation was noted with heat-stressed broilers fed diet containing triiodothyronine hormone (T3), where adding this hormone did not help to compensate its reduction in the broilers blood resulting from the heat stress (123). Also, adaptation was noted with broilers fed high-fiber diets and their reflection on liver fatty acids composition, where this composition was not affected (124).

However, different factors might affect carcass chemical composition, including, but not limited to, the amount of feed consumption, diet composition, AME:CP ratio, ambient temperature, and type and age of broiler chickens (86, 125–127). It seems that these effects might be noted only when the tested factor is used at high rate. However, the low level used of MPSM (10%) might be the reason for not getting an effect. Also, the equal energy and protein content in the two diets, used in this study, might played synergistic effect with the low level of MPSM for not getting differences in meat chemical composition among the different treatments.

Also, it was interesting to note that the glycogen contents did not decrease significantly in the groups that fed on 10% autoclaved MPSM with or without enzymes addition. These groups had lower acidity, meaning that they converted more glycogen to lactate; however, they still have a similar amount of glycogen to other groups. This might support the positive effect of autoclaving on the utilization of nutrients in the MPSM, and might be in the whole feed, as a result of reducing antinutrients (NSPs) in the MPSM. Similar findings were reported regarding the positive effect of autoclaving on laying hens’ performance (42).

### 4.6. Blood lipid profiles

It is well known that feed composition, medications, genetic factors, and enzymes addition all have a significant impact on blood lipid components (128, 129). Al-Harthi (47) found an increase in blood triglycerides and cholesterol values when olive cake (OC) inclusion increased from 0 to 10%, and (48) noted an increase in blood triglycerides, cholesterol, and total lipids values when OC increased from 0 to 15% in the diets of broilers between 1 and 28 d of age. The previous researchers attributed these effects mostly to the amount and composition of the soybean oil that was added to the feed to achieve the metabolizable energy requirements. These amounts of oil ranged from 3.6% in the control diet (0% OC) to 7.0% in 10% OC diet, and 8.8% in 15% OC diet. To a lesser extent, the researchers related these effects to the amount and composition of the remaining oil in the OC used, which ranged from 1.18% to 1.77% in the 10% and 15% OC diets, respectively. However, the findings of the current study indicated no significant differences on blood lipid components (triglycerides, cholesterol, total lipids), and this could be explained by the similar amount of oil added to the control and MPSM diets.

On the other hand, Al-Harthi et al. (42) suggested some negative effects of dietary fibers on lipids utilization. Yolk color score decreased when whole *Prosopis juliflora* pods meal (WPPM) was included in laying hens’ diets at 30% level, and fibers contents were 2.3 and 6.6% in the control and 30% WPPM diets, respectively, with a difference of 4.3%. The antinutritional agents contained in fibers increase viscosity, preventing the actions of endogenous enzymes, and they also decrease lipase and bile salt secretion, which, in turn, decrease dietary fat digestion and utilization (42, 92–94, 96, 130). Adding insoluble fibers (Arbocel RC Fine, JRS, Rosenberg, Germany; at 0%, 1%, and 2% levels) to the diet of young broilers between 7 and 21 d of age reduced abdominal fat, liver cholesterol, and blood cholesterol but did not affect total liver lipids, blood triglycerides, high and low-density lipoprotein, and fatty acids components (mg/g) of the liver (130). However, it seems that the effects of fibers on blood lipid components are dependent on the levels and types of fibers in the ingredient, the inclusion level of the ingredient in the feed, as well as the type and age of the animal used. The difference in fibers level between the two diets used in this study (1.7%) might be less effective to give an effect on blood lipid components.

On the other hand, using phytase enzyme as a single enzyme induced a decrease in the blood cholesterol and very-low-density lipoprotein (VLDL) values of broilers fed 5 and 10% levels of OC (47), as well as the blood cholesterol and triglycerides values decreased with 10 and 15% OC inclusion (48), when compared to their control groups who had the same inclusion levels of OC but without phytase addition. The reduction in the blood cholesterol, triglycerides, and VLDL values was attributed to the effect of phytase enzyme on lipids absorption and metabolism. The phytase enzyme, which was used in both studies (47, 48) is (Phyzyme XP, Danisco Animal Nutrition, Marlborough, United Kingdom, derived from *Escherichia coli*, and added at the level of 1 g/kg diet, which provided 10,000 FUT/kg diet). Phytase enzyme acts on the phytate component by releasing Zn, Mn, Cu, Se, Fe, Na, Ca, P, and Ca/P balance, which, in turn affect fatty acids absorption and lipids metabolism. Furthermore, it is interesting to focus on the composition of the multi-enzyme Galzym which was used in the same study (47). Galzym contains different enzymes, including the phytase enzyme (Galzym is a product of Tex Biosciences (P) Ltd., India, and it is a multi-enzyme containing cellulase 100,000,000 U/kg, xylanase 1,500.000 U/kg, lipase 10,000 U/kg, amylase 125,000 U/kg, protease 15,000 U/kg, pectinase 30,000 U/kg, arabinase 7,000 U/kg, phytase 200,000 U/kg, α-galactosidase 10,000 U/kg, and β-glucosidase 10,000 U/kg, and added at the level of 0.5 g/kg diet). However, although Galzym contains phytase enzyme, it did not produce the same effect on blood cholesterol and VLDL values as the single phytase enzyme did. To illustrate, Phyzyme XP, the single phytase enzyme that was used, provided 10,000 FTU/kg diet at an addition level of 1 g/kg diet. In contrast, Galzyme, the multi-enzyme, provided only 100 FTU/kg diet at an addition level of 0.5 g/kg diet. This can be calculated as follows: 0.5 g (the addition level) X 200,000 FTU/kg (the concentration of phytase enzyme per kg of the enzymatic mixture) /1,000 g = 100 FTU/kg diet. This might clarify the importance of the enzymes addition level for achieving an effect; thus, this promotes elevating enzymes addition levels to achieve their effects, especially when using ingredients that including high fibers level and consequently phytate content, as studies evidenced the association between fibers content and phytic acid as previously mentioned. As such, the effect of the phytase enzyme in the enzymatic mixture used in this study (Rovabio Advance PHY T) on the blood lipid components was not found, and this might be attributed to insufficient addition level, resulting in a shortage in the enzymatic power to match the complexity of the MPSM chemical composition, particularly regarding the amount of phytase enzyme. It provided 1,000 and 2000 FTU/kg diet based on the addition doses used (0.1 and 0.2 g/kg diet), and the phytase concentration in the enzymatic mixture is 10,000 FTU/g. It is very important to remember that MPSM, used in this study, includes high levels of fiber, NDF, ADF, and ADL. Again, different high doses of multi-enzyme that include all possible commercial enzymes should be used to test the possibility of achieving an effect, if found, on the MPSM by-product. It is worth mentioning that OC, which was used in the previous studies contained 14.1% crude fiber, which is lower than the fiber content in the MPSM (20.1%). Thus, this highlights the need to employ a dose of phytase more than the 2000 FTU/kg diet that was used in this study. However, the absence of the effects of multi-enzyme on blood hematology, protein and its fractions, and lipid components of broiler chickens were previously reported (45, 56). Also, EL-Hak et al. (4) ascertained no effect of different daily doses of MP dry seed, which were given orally to rats for 14 d, on albumin, total protein, insulin, creatinine, urea, uric acid, testosterone, follicle-stimulating hormone, and luteinizing hormone levels, while blood sugar, cholesterol, triglyceride, and liver enzymes levels decreased significantly. Again, it is worth remembering that the results among studies might differ dependent on the factors that the study was subjected to.

## 5. Conclusion

Based on the present findings, raw MPSM processed by cold press contains high crude protein (25.3%) and apparent metabolizable energy contents (3,060 AME Kcal/kg); however, the nutrients appear quite limited for the overall growth and performance of broilers between 1–35 d of age. The inclusion of 10% autoclaved MPSM in broilers’ diets had no negative impact on the birds’ final body weight and mortality rate, but the FCR and EBI values were still lower than the control group. Generally, carcass traits, meat quality, and blood lipids metabolism were unaffected by this level of autoclaved MPSM. Poultry producers should limit the inclusion level of raw MPSM in broiler chickens’ diets to less than 10%. Furthermore, it is of great importance to apply other techniques such as autoclaving, microwaving, boiling (with different temperature degrees and/or durations), soaking, fermenting, milling, and adding different exogenous enzymes with varying doses, or combining some of these methods to increase the utilization and/or the inclusion level of MPSM in broiler chickens’ diets. This is required to fully assess the biological value of MPSM as a feedstuff. On the other hand, it might be useful for commercial companies, if possible, to think about producing chemical substances that can deal with antinutritional agents, especially the second and third classifications previously mentioned in this study, by alleviating their adverse effects, and such substances can be added alongside commercial enzymes. Ultimately, this can help reduce time and cost compared to using traditional methods only.

## Data availability statement

The raw data supporting the conclusions of this article will be made available by the authors, without undue reservation.

## Ethics statement

This study was conducted at the Hada Al-Sham Research Station, Faculty of Environmental Sciences, King Abdulaziz University, Jeddah, Saudi Arabia. The research included two parts: The first part was the evaluation of MPSM chemical composition. The second part was the feeding experiment that was carried out on 280-1-d-old Ross-308 unsexed broiler chickens. For the feeding experiment, the methodology was verified by the Committee of the Agriculture Department, King Abdulaziz University, regarding the regulations of the scientific research ethics on living creatures. Which controls the rights and welfare of animals according to the Royal Decree No. M/59 dated 24/8/2010, and institutional approve code ACUC-22-1-2.

## Author contributions

MA-H: funding, conceptualization, methodology, project administration, investigation and approval, and writing final draft. MA-H and ME: following up the experimental work, data collection, and samples analysis. ME: software and data analysis. YA and FB: writing original draft. All authors contributed to the article and approved the submitted version.

## Funding

The authors respectfully recognize the Ministry of Education and King Abdulaziz University in Jeddah, Saudi Arabia, for financial support of this research study, which was funded by institutional fund projects under the grant number (IFPRC-200-155-2020).

## Conflict of interest

The authors declare that the research was conducted in the absence of any commercial or financial relationships that could be construed as a potential conflict of interest.

## Publisher’s note

All claims expressed in this article are solely those of the authors and do not necessarily represent those of their affiliated organizations, or those of the publisher, the editors and the reviewers. Any product that may be evaluated in this article, or claim that may be made by its manufacturer, is not guaranteed or endorsed by the publisher.
